# A Review on Therapeutic Effects of *Labisia pumila* on Female Reproductive Diseases

**DOI:** 10.1155/2021/9928199

**Published:** 2021-09-15

**Authors:** Alif Aiman Zakaria, Mohd Hezmee Mohd Noor, Hafandi Ahmad, Hasliza Abu Hassim, Mazlina Mazlan, Mohd Qayyum Ab Latip

**Affiliations:** ^1^Department of Veterinary Preclinical Sciences, Faculty of Veterinary Medicine, Universiti Putra Malaysia, 43400 Serdang, Selangor, Malaysia; ^2^Department of Veterinary Pathology and Microbiology, Faculty of Veterinary Medicine, Universiti Putra Malaysia, 43400 Serdang, Selangor, Malaysia

## Abstract

The *Labisia pumila* (LP) is a traditional plant that is locally known as Kacip Fatimah, Selusuh Fatimah, or Pokok Ringgang by the Malaysian indigenous people. It is believed to facilitate their childbirth, treating their postchild birth and menstrual irregularities. The water extract of LP has shown to contain bioactive compounds such as flavonoids, ascorbic acid, *β*-carotene, anthocyanin, and phenolic acid, which contribute extensive antioxidant, anti-inflammatory, antimicrobial, and antifungal. The LP ethanolic extract exhibits significant estrogenic effects on human endomentrial adenocarcinoma cell in estrogen-free basal medium and promoting an increase in secretion of alkaline phosphate. Water based has been used for many generations, and studies had reported that it could displace in binding the antibodies and increase the estradiol production making it similar to esterone and estradiol hormone. LP extract poses a potential and beneficial aspect in medical and cosmeceutical applications. This is mainly due to its phytoestrogen properties of the LP. However, there is a specific functionality in the application of LP extract, due to specific functional group in phytoconstituent of LP. Apart from that, the extraction solvent is important in preparing the LP extract as it poses some significant and mild side effects towards consuming the LP extracts. The current situation of women reproductive disease such as postmenopausal syndrome and polycystic ovary syndrome is increasing. Thus, it is important to find ways in alternative treatment for women reproductive disease that is less costly and low side effects. In conclusion, these studies proven that LP has the potential to be an alternative way in treating female reproductive related diseases such as in postmenopausal and polysystic ovarian syndrome women.

## 1. Introduction

The *Labisia pumila* (LP) is a traditional plant that is commonly known as Kacip Fatimah, Selusuh Fatimah, and Pokok Ringgang by the indigenous people. It is believes to facilitate during a childbirth, treating their postchildbirth and menstrual irregularities [1]. LP is plant grown in low cluster, and it has rarely branching stems with a hairy root. The oblong shape of LP can grow up 20-40 cm [2]. The most common form of consuming this LP is through decoction, and it is believed to improve their reproductive health and sexual lifestyle. In the last decade, there have been many debates on the safety and efficacy use of LP on human. Modern pharmacologists have done experimental investigation on the properties of LP which they possess antioxidant, antimicrobial, anticancer, and anti-inflammatory, and LP can exert phytoestrogenic effects [3, 4]. However, it is important to understand the compound in LP that contributes to the health benefits on reproductive performance in women.

The water extract of LP has shown bioactive compounds such as flavonoids, ascorbic acid, *β*-carotene, anthocyanin, and phenolic acid which contribute extensive antioxidant, anti-inflammatory, antimicrobial, and antifungal [5]. The more superior antioxidant activity in LP is related to high in phenolic and flavonoid compound [6]. Studies by Effendi and Shuid [7] shown that LP can exert high level of antioxidant enzymes and reduced the malondialdehyde level, which in turn regulate the lipid peroxidation and marker to oxidative stress. Furthermore, the growing interests in LP on phytoestrogen effects have shown potential benefits on the women reproductive performance. This phytoestrogen in LP acted as a pair of hydroxyl group and a phenolic ring which function to bind estrogen receptors [8]. Thus, the binding process of phytoestrogen to estrogen receptor may exert the estrogenic and antiestrogenic effects [9]. The phytoestrogen LP has proven to improve in health benefits on postmenopausal women and facilitate in childbirth. Apart from that, different extraction of LP also plays a major role on the phytoestrogen. It was also proven that the ethanolic extract is able to increase a significant estrogenic effect towards human endometrial adenocarcinoma cells [10].

The estrogen deficiency has become a major problem among women. It is the cause of postmenopausal bone loss and contributes to the age-related bone loss. The lack of estrogen also results in both long-term and short-term effects. The short-term effect may cause a variable degree of vasomotor instability, fatigue, mood disturbance, and sexual dysfunction [11]. This is due to the onset of hypothalamo-pituitary disease or surgery. However, the long-term effects of estrogen deficiency are the reduction of bone mineralization leading to an increasing risk of bone fracture. Studies had shown that the estrogen deficiency is associated with the increase in production of tumor necrosis alpha factor by bone marrow T cells which augments receptor activator of nuclear factor kappa-B ligand (RANKL) induced by osteoclastogenesis [12]. Estrogen can induce osteoclast apoptosis and inhibit osteoblast apoptosis which will indirectly reduce the bone resorption and increase the bone formation activity (S N [13]). Estrogen is able to suppress production of cytokine such as IL-1, IL-6, and TNF-ɑ [14]. LP that is known to exert phytoestrogenic effect can be used as an alternative to ERT that can produce positive effects on bone without causing side effects. LP contains antioxidant as well as exerting anti-inflammatory effect, which can act as free radical scavenger, thus inhibiting TNF-*α* production and COX-2 expression that leads to a decline in RANKL expression.

The administration of LP can be an alternative to synthetic estrogen for hormone therapy to reduce side effects of prolonged hormone therapy such as risk of breast cancer, endometrial cancer, and cardiovascular diseases [14, 15]. However, there are also studies on herbal plants that result in inconsistent efficacy [3]. Thus, the objective of this review article is to demonstrate the inclusive studies on the therapeutic effects of LP on female reproductive related disorder and supported the use of LP as herbal medicine.

## 2. Properties of *Labisia pumila*

### 2.1. Antioxidant Properties of LP

Numerous studies had reported the health benefits of LP on the prevention and treatment of diseases related to estrogen deficiencies. The antioxidant property of LP is one of the factors that contribute to the health benefits. Currently, various types of phytochemicals had been identified by the extraction of LP, and most possess beneficial properties and are consistent in their traditional uses. The LP extract mainly contains flavonoids, polyphenols, phenolic acids, and saponins [6]. Many researches had suggested that total flavonoid is important secondary metabolite that promotes strong bioactivity such as free radical scavenging, antioxidant, anti-flammatory, and anticancer [16]. The total flavonoid mainly composes of flavonoids, isoflavones, flavanols, isoflavanones, and dihydroisoflavones. The parent compound of flavonoids is quercetin, myricetin, kaempferol, rutin, protocatechunic acid, catechin, naringin, daidzin, gegnistein, and anthocyanin [17]. It was proven that total flavonoids had been effectively scavenging the free radicals of most types of oxidizing molecules, including the singlet oxygen and various free radicals [18].

The LP is inclusive to possess many antioxidant properties due to the abundance of these phytochemicals. Protecting against many chronic diseases and delaying aging process are some to the key role of plant antioxidants. On the other hand, phenolic compound is found in most plants, and this compound is known to possess potent antioxidant activity and has anticancer, antibacterial, and anti-inflammatory properties to a greater or lesser extent [19]. Studies had shown that LP contains a significant amount of phenolic compound with the amount of value ranging from 2.53 to 2.55 mg/g throughout the different species of LP [4]. However, LP has received wide attention on its phytoestrogenic effect on women. [1] show that anthocyanin in LP is high compared to other phytoconstituents. More studies are needed to prove on the phytoestrogenic properties of each phytoconstituent in LP, perhaps on the specific phytoconstituent of LP such as anthocyanin which promotes benefits on cardiovascular diseases. However, extraction methodology needs to be confirmed on specific phytoconstituents of LP.

Some studies showed LP could help in reducing the oxidation process in postmenopausal syndrome by maintaining the superoxide dismutase in the postmenopausal syndrome model [7]. This can promote the oxidation process by reducing the free radical that is harmful to the body. It is also believe that LP is said to be able to repair oxidative damage (S N [13]).

Studies from Nurdiana et al. [20] reported on oxidative stress on rat model of postmenopausal osteoporosis. The study group of overiectomized was treated with LP (10 mg/kg, 20 mg/kg, and 40 mg/kg). The treatment was administered for 8 weeks. The results showed that the levels of serum malondialdehyde were significantly reduced by the treatment of LP reaching the levels comparable to the control group. The serum levels of superoxide dismutase were significantly higher in the group of overiectomized rats compared to the control group. This showed that the administration of 20 mg/kg treatment of LP significantly lowered the superoxide stress relative to group of overiectomized control. The administration of 20 mg/kg of LP had given the best result on oxidative stress. It was found that the administration of 40 mg/kg did not give any good result. This could be that the administration of 40 mg/kg treatment of LP was toxic to the overiectomized rat model. However, further study is needed to confirm the toxicity effect of LP dosage.

### 2.2. Phytoestrogenic Properties

Phytoestrogen is a compound that is naturally found in plants. They are found in a wide range of plant-based foods. A plant-based diet is very rich in natural phytoestrogen in healthy amounts that can promote health benefits towards women who are lacking in estrogen hormone. In recent years, the application of LP as an alternative replacement for estrogen replacement therapy has become popular. This is due to its phytoestrogenic activity. It is said that the phytoestrogen has a pair of hydroxyl group and phenolic ring which is required for binding of estrogen receptors [21]. Furthermore, the water extract of LP that has been used for many generations could inhibit estradiol binding to antibodies raised against estradiol, which is showing similar effect as estrone and estriol [22]. However, Jamal et al. found that different part of LP exhibited different form of estrogenic activity. The ethanol extract of LP roots showed a weak estrogenic activity at 10 to 50 mg/ml in an in vitro assay in Ishikawa cells, but none was observed in leaf extract. Moreover, no estrogenic activity was seen in the aqueous extract from the roots of LP [10].

The LP ethanolic extract exhibited significant estrogenic effects on human endomentrial adenocarcinoma cell in estrogen-free basal medium and promoting increase secretion of alkaline phosphate [23]. Water based has been used for many generations, and studies had reported that it could displace in binding the antibodies and increase the estradiol production making it similar to esterone and estradiol hormone [24]. In the study of postmenopausal syndrome disease, the intake of phytoestrogen in plant was found to be associated with lower aortic stiffness. The phytoestrogenic properties of LP were reported having the ability to maintain elastic lamella structure of aorta in overiectomized rats. Postmenopausal syndrome had been linked to cardiovascular diseases due to lack of estrogen hormone. The aortic stiffness consequently leads to the risk of cardiovascular disease in postmenopausal syndrome [20].

Phytoestrogen possesses wide estrogenic biological properties and is self-prescribed as safe for dietary supplement for postmenopausal women. In the present studies, LP was shown to have the ability to treat osteoporosis in postmenopausal women. It is believed that isoflavones of soy, genistein, have been shown to preserve bone and prevent bone loss of bone mineral density of overiectomized rat model [25]. The understanding of phytoestrogen properties of LP can promote a better management of osteoporosis in postmenopausal women.

### 2.3. Anti-Inflammatory Cytokine Properties

Many experts suggest that anti-inflammatory diet may help in reducing the inflammation like increase in adipose tissue and osteoporosis in postmenopausal women. The increase in risk of inflammation is due to the lack of estrogen hormone in women. Various epidemiological studies have shown the risk of developing osteoporosis in an inflammatory condition such as rheumatoid arthritis, haematological disease, and inflammatory bowel disease [26]. LP has been proven to have the anti-inflammatory properties from the recent research. Proinflammatory cytokines such as tumor necrosis factor- (TNF-) *α*, IL-6, IL-1, IL-11, IL-15, and IL-17 are elevated in these conditions [27]. Studies suggested that they promote osteoclast differentiation which acts as a stimulator for bone resorption and has been linked to bone loss seen in postmenopausal women (Siti Noor [28]). The receptor activator of NF-*κβ* ligand (RANKL) is a membrane-bound molecule of TNF ligand family which functions in the osteoclasts formation [29].

The involvement of TNF-*α* cytokine in inflammation is an important cofactor in bone resorption. This is due to the fact that cytokine supports osteoclast activation mediated by RANKL and macrophage colony-stimulating factor. However, estrogen is able to suppress the production of this TNF-*α* [30]. This is why estrogen withdrawal following menopause will lead to an increase in these cytokines as proven in many studies. Studies on bone resorption demonstrated that the fall of estrogen level in postmenopausal women was able to stimulate local inflammation in the bone, leading to a decrease in bone density.

## 3. LP Increase Estrogen of Postmenopausal Syndrome

Women are considered to be postmenopausal when they have not had their period for an entire year. Postmenopausal syndrome has been linked to many health conditions such as osteoporosis and cardiovascular diseases. This is mainly due to the decrease in the production of estrogen hormone. The consumption of LP water extracts was found to be able to displace estradiol binding to antibodies raised against estradiol, making it similar to other estrogen. The water extract of LP has been found to produce dose response effects on reproductive hormone of female rats such as estradiol and free testosterone levels [31]. Among the study of LP on postmenopausal women by Norhayati et al. [11], LP water of 400 mg was given to postmenopausal women at the age of 40–60 years. A women health questionnaire was used to assess the quality of life. Women with postmenopausal showed improvement in memory and concentration (8.3%), vasomotor symptoms (15.9%), menstrual symptoms (11.8%), and sleep problems (31.0%). Improvement was shown in cardiovascular parameters; however, LP showed no changes in luteinizing hormone, follicle-stimulating hormone, and 17*β*-estradiol.

In the study of body weight gain of OVX rats, 30 days of treatment of OVX rats with LP 50 mg/kg/day significantly lower the body weight gain compared to untreated OVX rats [32]. It is also observed that treatment of LP extract increases the uterine weight of OVX rats given with 10, 20, and 50 mg/kg/day for 30 days of treatment [32]. However, there was no significant change in the plasma adiponectin level observed in OVX rats treated with LP extract [32]. In a study of LP extract treatment of 280 mg/kg for 6 months in postmenopausal women, it was reported that LP extract was able to lower the triglyceride value of postmenopausal women compared to the placebo group [33]. However, the study showed no hormonal change in the treatment of LP extract on postmenopausal women. In animal study using overiectomized rats, LP plant extract was found to be able to increase the estradiol level and suppress the follicle-stimulating hormone (FSH) and luteinizing hormone (LH) which gave the same effect as hormonal replacement therapy [15]. It is believed that LP extract acts as selective estrogen receptor modulator that is partially antagonist [34].

Postmenopausal women have become a primary public health problem. One of every three women above 50 years old was reported to experience fracture related to osteoporosis [35]. 8.9 million fractures (hip: 1.6 million, forearm: 1.7 million, and clinical vertebral: 1.4 million) were recorded annually, and 61% were from women [7]. Furthermore, consumption of hormonal replace therapy (HRT) has been linked to many debilitating effect towards the postmenopausal women. This HRT has been linked to breast, colorectal, and endometrial cancer, and LP extracts have been used as an alternative treatment for postmenopausal women due to its phytoestrogenic effects.

To date, there is no thorough study on mechanism of action for LP on postmenopausal syndrome and PCOS. However, LP works can be speculated through estrogenic pathways. An increase in the resistin level is consistent with i*n vitro* studies of adipocytes which shows similarity to estradiol in stimulating resistin gene expression [36]. The plant root and leaves contain two novel compounds which are benzoquinoid compounds 1 and 2, as major compound [33]. Phytoestrogens in LP exert their effect through estrogen receptor [37]. Thus, able to replace the estradiol binding to antibodies makes it similar to estrone and estradiol [37]. However, the effect of LP extract might take a longer time on dose response effect compared to HRT. The HRT might take 30 days to increase estradiol level while LP extract may take 60 days of treatment [15].

## 4. Effect of LP on Polycystic Ovary Syndrome (PCOS)

The polycystic ovary syndrome (PCOS) is a disease that affects women's hormonal levels. Women with PCOS produce higher amount of male hormones called androgen compared to normal women. This hormonal imbalance causes women with PCOS to skip their menstrual periods, and it is hard for them to get pregnant. One of the main reasons for PCOS development is obesity. It has been linked to many metabolic and reproductive disorders including PCOS [38]. PCOS is a common endocrine disorder affecting to 10% of women in their reproductive age, and it is reported that the 40%–80% of PCOS are experiencing obesity [39]. The key characteristic to determine whether they have PCOS is the presence of two of the three symptoms of hyperandrogenism, chronic anovulation, and polycystic ovaries [40]. However, PCOS tends to present many metabolic disorders such as weight gain, hyperinsulinemia, insulin resistance, and abdominal adiposity [41]. Furthermore, it is suggested that the increase in insulin levels plays a key role in ovarian androgen production, and hyperinsulinemia may cause the development of PCOS.

Many researches were using androgen treatment to induced PCOS in rodents. However, this model does not produce key metabolic feature of PCOS such as hyperinsulinemia. High-fat diet (HFD) has been used to induce PCOS in rodent's models [42]. HFD was found to cause obesity and cause metabolic disturbance such as hyperinsulinemia and insulin resistance. A study by Roberts et al. [42] had shown the development of PCOS on the rodent model given with HFD with the key characteristic of metabolic disturbance, polycystic ovaries, and irregular estrous cycle. It had also shown that the elevated testosterone was observed in the rodent model given with HFD. Furthermore, this option in using HFD to develop PCOS may be the best method to simulate the PCOS in human.

The current treatment for PCOS is Metformin, which helps in insulin resistance by increasing the insulin sensitivity of PCOS. However, there are long-term effects to this treatment such as diarrhea and impaired renal function. Progestins were used to treat the hyperandrogenism. However, this drug promotes weight gain, fluid retention, and liver dysfunction. Alternative treatment is seen to be an option to treat PCOS such as LP extracts. Study had shown that LP extract can increase the uterine weight and increase insulin sensitivity without affecting the body weight in PCOS rat model [43]. The LP extract had also shown to improve the lipid profile of PCOS by reducing the total cholesterol and triglycerides levels in PCOS rat models. There are not many researches on LP extract on PCOS treatment currently. However, LP extract can be an alternative treatment for PCOS due to its low cost and safe for human consumption. Further study is needed in the treatment of PCOS with LP extract.

## 5. Effect of LP on Bone Density and Bone Strength on Osteoporosis

Osteoporosis is a skeletal disease that causes low bone mass and microarchitectural deterioration. The effect of low bone mass increases the tendency of bone fracture and fragility. The common type of bone fracture is osteoporotic fracture on hip, wrist, and vertebral bones. The tendency of women to get osteoporosis has increased tremendously in the last five decades which accounts on 17.5% in women and 6% in men [44]. The osteoporosis occurs when bone mineral density falls to more than 2.5 standard deviation below the standard reference of maximum bone mineral density of young adult female [45]. Also, women were found to have higher tendency of having osteoporosis than men in ratio of 1.6 : 1 which is mainly due to postmenopausal syndrome in women [46]. However, in PCOS-related disease, women tend to develop estrogen deficiency that will affect the bone health. A study by [47] showed that PCOS tends to develop lower bone mineral density (BMD) values compared to the control group (1.057 ± 0.1260 vs. 1.210 ± 0.1805 g/cm^2^, *p* < 0.0002). Furthermore, the study suggested that BMD is correlated to insulin concentration in PCOS women. In much severe cases of PCOS, a complex endocrine disorder and metabolic disturbance can lead to osteoporosis.

Estrogen hormone in women protects the bone loss that can lead to osteoporosis. This estrogen deficiency can be treated by estrogen replacement therapy (ERT) such as premerin. ERT has been proven to increase the lumbar spine and proximal femur [35]. It also can be given in combination with progesterone to increase the antiresorptive effects on bone cells affecting the osteoclast activity [35]. However, this ERT can lead to many side effects such as breast cancer and endomentrial cancer (Poh Su Wei [48]). Studies had shown that giving LP water extract of 100 mg/kg to overiectomized rats can be as good as ERT supplementation [47]. Furthermore, LP water extract of 17.5 mg/kg given to overiectomized rats had proven to increase the osteocalcin levels compared to the control group and prevented any change in bone biochemical markers [46]. However, supplementation of LP water extract is unable to prevent bone calcium content loss in overiectomized rats [46].

Bone remodeling can be categorized as bone formation and bone resorption. There are several factors or cytokines known to play the important roles in bone remodeling. Bone resorption is regulated by receptor activator of nuclear factor kappa-B ligand (RANKL) and Osteoprotegerin (OPG), which are produced by osteoblasts. RANKL binds to RANK receptors which are located on osteoclast precursors to promote differentiation into mature osteoclasts and activate their bone resorptive activity. Studies had reported that consumption of LP water extract protects the bone from estrogen deficiency by regulating the RANKL, Osteoprotegerin, and bone morphogenetic protien-2 of gene expression which can lead to osteoporosis (S N [13]).

## 6. Fat Metabolism

### 6.1. Reduce in Fats and Improvement in Cardiovascular Disease in Postmenopausal Women

Body weight is a common form of physiological changes in women experiencing estrogen deficiency, a significant gain in fat mass in postmenopausal women specifically on adipose tissue. The lack of estrogen hormone influences the amount of fat in the body which regulates the glucose and lipid metabolism. Research suggested that women approaching menopause tend to become overweight. However, in PCOS-related disease, it had been reported that there was an increase in weight gain results from obesity [42]. The underlying cause is due to the overconsumption of HFD that has disturbed the glucose and lipid metabolism. Adipose tissue which significantly increases due to the lack of estrogen is an endocrine organ that regulates the signaling molecules such as adipokines that promote the growth factors, cytokines, and complement factors [32]. The main type of adipokines is leptin, resistin, and adiponectin. The dysregulation of this hormone has led to the increase in visceral fats. The visceral fats play an important role in pathogenesis of insulin resistance, hypertension disorder of coagulation, and metabolic syndrome which can be seen in PCOS and postmenopausal women ([49].).

### 6.2. Mechanism of Fat Metabolism

One of the key associates to obesity is the increase in leptin concentration in relation to fat mass. Leptin is the 167-amino acid which is produced by the hypothalamus, and it is involved in the regulation of energy homeostasis, neuroendocrine function, and metabolism of energy deficiency [50]. The leptin is secreted by the adipose tissue, and the amount of leptin produce is correlated to the amount of body fat in the body [51]. In the current situation, some studies suggested that an early exposure of formula feed or feeding with high fat diet during infancy increases the leptin concentration in later life. The increase in leptin during infancy thus increases the prevalence of obesity.

**Figure 1:**
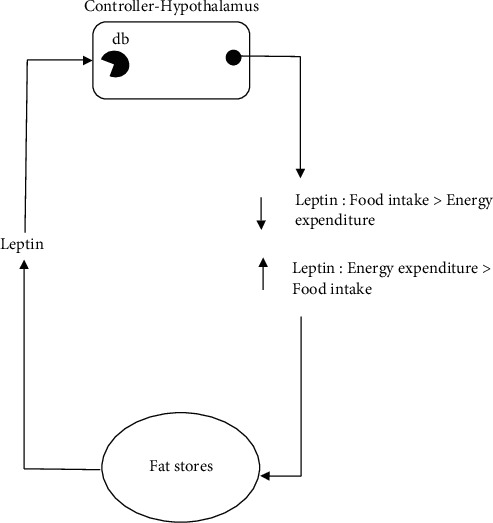
Mechanism of leptin production in human body [54].

In the prevalence of overweight during infancy, experimental data on rat models supported the hypothesis. Rats that were food restricted before weaning were permanently lighter regardless of how much food available after weaning [52]. It is important to understand the mechanism of how leptin works. Leptin was first discovered by Douglas Coleman in 1970 when he studied the obese and diabetes mice. It was said that the recessive mutation in the mouse of obese and diabetes genes resulted in obesity and diabetes syndrome resembling the morbid human obesity [53].  Figure 1 shows the mechanism of leptin that is produced by the stored adipose tissue under the homeostatic control. The secretion of leptin is stimulated by the hypothalamus in the brain caused by the energy deficiency in the body. The level of leptin is positively correlated with differences in body fat, where an increase in leptin results in negative energy balance [54]. Leptin reduced food intake and increased energy expenditure in obese mice, and it also corrected the endocrine abnormalities of obese mice and restored reproductive performance of obese mice [55]. Studies also suggested that leptin reduces the weight in all mammalians [56]. In fact, in most obesity cases, they are most often being associated with high level of leptin. This may be due to the leptin resistance that is caused by long exposure of high-fat diets.

The leptin circulating in the body is found higher in concentration level in women compared to men. The leptin level is highly associated with the amount of adipose tissue in the body. There is a correlation between the tissue lipid content and the gene expression, and they are the same in human and in rodents [57]. These differences are due to the attribution of adipose tissue, which are more on abdominal in men and mainly subcutaneous in women. Women exhibit higher level of leptin compared to men, probably because of the different physiology regulatory effect of male sex hormone [58]. However, some data suggested there is no significant difference in the level of sex hormone in male and female Sprague Dawley rats [59], while other author found a significant higher leptin level in male compared to female Long-Evans rats [60].

### 6.3. Effect of LP Extracts on Fat Metabolism

A study by Fazliana et al. [32] reported that LP extract possesses some forms of fat metabolism by initiation of lipolysis. The study was done on overiectomized rats administered with LP 10 mg/kg and 50 mg/kg and compared with ERT. ERT administration decreased the body weight gain in OVX rats significantly while LP 10 mg/kg and 50 mg/kg prevented further weight gain in OVX rats. In a different study on women with postmenopausal syndrome, Abdul Kadir et al. [33] reported that the administration of LP water extracts of 280 mg/day showed the levels of triglycerides was significantly lowered by 1.4 mmol/L compared to the placebo 1.9 mmol/L after a six-month administration. However, the values of the triglycerides were in normal range, which has no clinical relevance. Furthermore, a study of clinical obese women for weight management and fatigue by Ahmad and Safuan [38] reported that administration with 125 mg twice daily for 40 days showed a favorable weight loss, with hip and waist circumference reduction. It also stated that the general quality of life had improved over the course of 40 days.

The effect of LP water extract on a body weight-reduction mechanism is still unclear. However, many reports had suggested that the mechanism was mainly attributed to the improvement of serum lipid profile, regulation of insulin sensitivity, regulation of oxidative stress, and inhibition of leptin obese model [61]. The phytoestrogenic activity of LP plays a role in regulating the adiposity of obese women. It has also been shown that the phytoestrogen can suppress the adipose tissue differentiation on lipid accumulation [62]. Previous study suggested that the LP oral administration was associated with the reduction of LDL levels and elevation of HDL levels in postmenopausal women [63]. Not only the consumption of LP has the potential in reducing body weight, and there was no report on any significant toxicity or increase in mortality. Thus, it is suggested that the LP water extract has the potential of lowering the body weight of obese women without any adverse effect.

## 7. LP on Skin Elasticity

In the modern era of science and burst of technology, plastic surgery and laser rejuvenation have become popular. There is a growing tendency of people in the 21^st^ century trying to look younger by taking care of their skin. The chronic exposure of skin towards the sun will cause the skin to age even faster which is known as photoaging. This is due to the ultraviolet (UVB) irradiation that promotes premature aging characteristics such as wrinkles, roughness, laxity, and pigmentation [1]. Interestingly, the aging process can slow down or even be reversed which is a time-dependent series [64]. However, human has to cope with the endogenous generation of reactive oxygen species which is the product of physiological cellular metabolism which eventually degraded the skin aging [1].

LP water extracts had been reported to have antiaging characteristic due to its antioxidant properties. A study by [65] reported that 50% of free radical scavenging activity (FSC_50_) of LP water extracts was determined to be 0.006%, which was the same as giving 156 *μ*M of ascorbic acid. The decrease in collagen synthesis human fibroblast by the ultraviolet light was restored to normal level by the treatment with LP water extracts. This is mainly due to the presence of bioflavonoid and phenolic acid content in the LP extracts [6]. The quercetin is one of the major components in the LP water extracts [66]. It is believed that quercetin has cytoprotective property against oxidative stress. This compound has the rejuvenating effects that reduce the tyrosinase which would provide a whitening effect on the skin. Therefore, LP water extract possesses the potential as an alternative treatment for aging on the skin and ingredients for cosmeceutical formulation.

## 8. Side Effects of LP Extracts

There is still very little information on the toxicity study of LP extracts towards human consumption and medical application. However, in Malaysia, most traditional practitioner will recommend LP extracts for treatment of flatulent, dysentery, and postpartum herbs. Effendy et al. (2010) conducted a study on the effect of LP petroleum ether extract on liver and kidney of white rats. The rats were subjected to subcutaneous injection of LP petroleum ether extract at 0.1, 0.05, and 0.025 mg/ml, respectively. The liver and kidney of the rats showed an abnormality in tissue degenerative process. This indicated that there was a presence of toxin in the compound of LP's petroleum ether extracts. Apart from that, however, there were many reports that LP water extracts are safe for human consumption. The Institute of Medical Research, Malaysia [67], suggested that cytogenetic of toxicology of LP in bone marrow of Sprague Dawley rats showed micronucleus frequencies that could be considered to be reliable index for detecting chromosome breakages. There is no significant increase in the micronucleated polychromatic erythrocytes when administered with 100, 700, and 2000 mg/kg of rat body weight of LP extracts. Besides, there was no report on dose effect on the rat consumption [1].

Regarding the teratogenicity and reproductive toxicity study of LP extracts, University Sains Malaysia had evaluated the LP water extracts and did not find any significant teratogenic toxicity effect after administered with up to 1000 mg/kg towards Sprague Dawley rats (Wan Ezumi [68]). The toxicity level was also tested in the estrous cycle and reproductive performance of postnatal growth and the offspring survival of rats (Wan [69]). The results also had proven to be not significant in reproductive toxicity during pregnancy. The received LP extracts were up to 800 mg/kg of rat body weight.

Finding the ways of linking estrogen use for treatment in women disease can be beneficial in the future. The LP extracts can pose none or minimal side effects towards human consumption. This will in turn replace the estrogen replacement therapy which poses detrimental effects towards women. However, more studies are needed in order to confirm the effectiveness and toxicity safety of LP extracts.

## 9. Discussions and Conclusion

Based on the review, the LP extract poses potential and beneficial aspects in both medical and cosmeceutical applications. This is mainly due to its phytoestrogen properties of the LP. However, there is specific functionality in application of LP extract, which might be due to specific functional group in phytoconstituent of LP. Apart from that, the extraction solvent is important in preparing the LP extract as it poses some significant and mild side effects when consuming the LP extracts. Due to its great demand and significant beneficial effects towards women, researches have been carried out worldwide. This is to ensure that the benefit of LP extract can be commercialized and benefits those in need.

The article reviews the current studies of LP extracts especially on women reproductive disease. The benefits of this plant pose phytoestrogenic properties and also antioxidant and antimicrobial are in line with the traditional use in postpartum medication. It is also proven to be beneficial in skin aging process as it can upregulate the synthesis of collagen in the skin fibroblast. It is important to note that the LP extract can be used as an alternative treatment for osteoporosis disease. The current situation of women reproductive disease such as postmenopausal syndrome and polycystic ovary syndrome is on the increase. Thus, it is important to find alternative treatment for women reproductive disease that is less costly and low side effects. In conclusion, these studies had proven that LP has the potential to be an alternative way in treating female reproductive related disease such as in postmenopausal and polysystic ovarian syndrome.
